# Hemorrhagic pericardial effusion resulting in constriction in hereditary hemorrhagic telangiectasia

**DOI:** 10.1186/s13019-022-01782-1

**Published:** 2022-03-21

**Authors:** Joshua S. Chung, Ryan Bylsma, Laura J. Denham, Huayong Hu, Nirav Mamdani, Aditya Bharadwaj, David G. Rabkin

**Affiliations:** 1 Department of Cardiothoracic Surgery, Loma Linda University Medical Center, Loma Linda, CA USA; 2 Department of Pathology, Loma Linda University Medical Center, Loma Linda, CA USA; 3 Department of Anesthesia and Critical Care Medicine, Loma Linda University Medical Center, Loma Linda, CA USA; 4 Division of Cardiology, Departments of Medicine, Loma Linda University Medical Center, Coleman Pavilion, Suite 21121, 11175 Campus Street, Loma Linda, CA 92354 USA

**Keywords:** Osler–Weber–Rendu disease, Pericardial constriction, Extra-corporeal membrane oxygenation

## Abstract

**Background:**

We report the first ante-mortem diagnosis of hemorrhagic pericardial effusion in hereditary hemorrhagic telangiectasia resulting in constriction; the case also demonstrates the unusual but well-described complication of right-sided heart failure requiring extracorporeal membrane oxygenation (ECMO) support after pericardiectomy.

**Case presentation:**

A previously healthy 48 year old man with a strong family history of Osler–Weber–Rendu disease presented to our institution with signs and symptoms of advance heart failure. His workup demonstrated a thickened pericardium and constrictive physiology. He was brought to the operating room where old clot and inflamed tissue were appreciated in the pericardial space and he underwent complete pericardiectomy under cardiopulmonary bypass. Separation from bypass, hampered by the development of right ventricular dysfunction and profound vasoplegia, required significant pressor and inotropic support. The right heart dysfunction and vasoplegia worsened in the early postoperative period requiring a week of ECMO after which his right ventricle recovered and he was successfully de-cannulated.

**Conclusion:**

Given the poor outcome of severe postoperative right ventricular failure after pericardiectomy, with high central venous pressure, a low gradient between central venous and pulmonary artery pressures and high vasopressor requirements, ECMO should be instituted promptly.

## Background

Originally labeled Osler–Weber–Rendu disease after the clinicians who first described the condition, hereditary hemorrhagic telangiectasia (HHT) is characterized by the primarily-dominant, autosomal transmission of dermal, mucosal and visceral telangiectasias and visceral arteriovenous malformations [1]. Spontaneous hemorrhage into the pericardium in the context of HHT is an unusual cause of pericardial constriction with only one reported case where the diagnosis was made post-mortem [2]. We report the first ante-mortem diagnosis of hemorrhagic pericardial effusion in HHT resulting in constriction; the case also demonstrates the unusual but well-described [3] complication of right-sided heart failure requiring extracorporeal membrane oxygenation (ECMO) support after pericardiectomy.

## Case presentation

A previously healthy 48-year-old male presented to our Emergency Department with dyspnea. Over the previous six months he had gained 60 pounds. He was unable to ambulate more than twenty-feet without resting. Although he had no history of epistaxis, mucocutaneous telangiectasia or documented visceral arteriovenous malformations, his family history was notable for a mother, two brothers and a daughter with genetically-confirmed HHT.

**Fig. 1:**
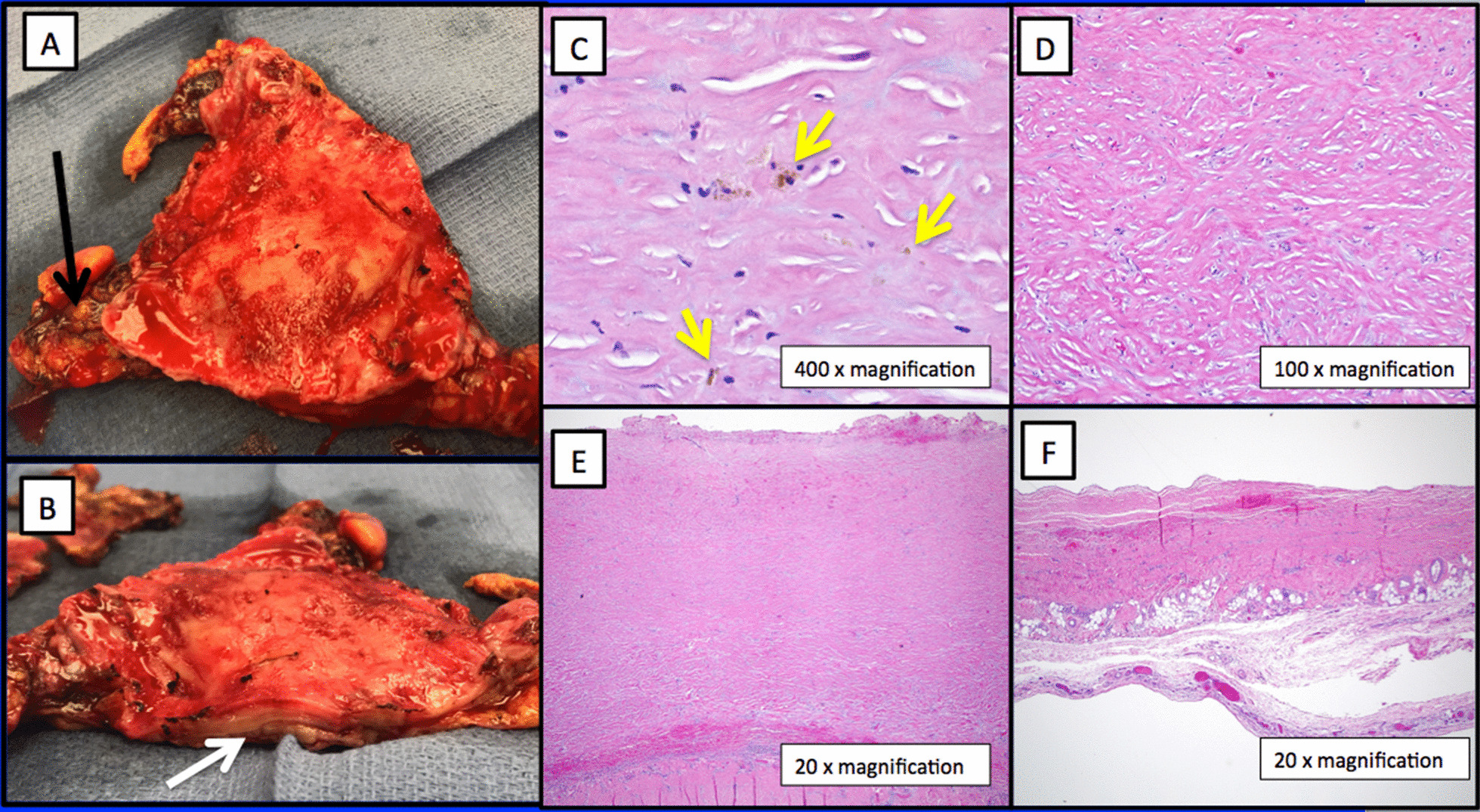
**a** Computed tomography demonstrating thickened pericardium (yellow arrow), pleural effusions and lung consolidation. Transthoracic apical four-chamber echocardiogram demonstrating elevated medial E’ velocity (**b**), septal movement towards LV on inspiration (yellow arrow) (**c**) and toward RV on expiration (red arrow) (**d**)

He was afebrile and hemodynamically stable. He had a rapid, regular heart rate, diminished breath-sounds bilaterally, pitting edema to his chest wall, a normal creatinine (1.0 mg/dL) and elevated total bilirubin (2.2 mg/dL). Viral and antibody testing for COVID-19 were negative. His chest roentgenogram demonstrated an enlarged cardiac silhouette, pulmonary interstitial edema and moderate-sized pleural effusions. Computed tomography demonstrated thickened pericardium and bilateral pleural effusions (Fig. 1a). An echocardiogram demonstrated normal biventricular function, thickened pericardium, severely elevated right atrial pressure with a dilated and non-collapsible inferior vena cava, elevated medial e’ tissue velocity of 12.6 cm/s (Fig. 1b) and pronounced ventricular septal bounce (Fig. 1c, d). Simultaneous pressure-tracings of the left ventricle (LV) and right ventricle (RV) and of the LV and wedge-pressure were pathognomonic of constrictive physiology. His coronary anatomy was normal. He was optimized on the medical service before going to the operating room for radical pericardiectomy.

**Fig. 2:**
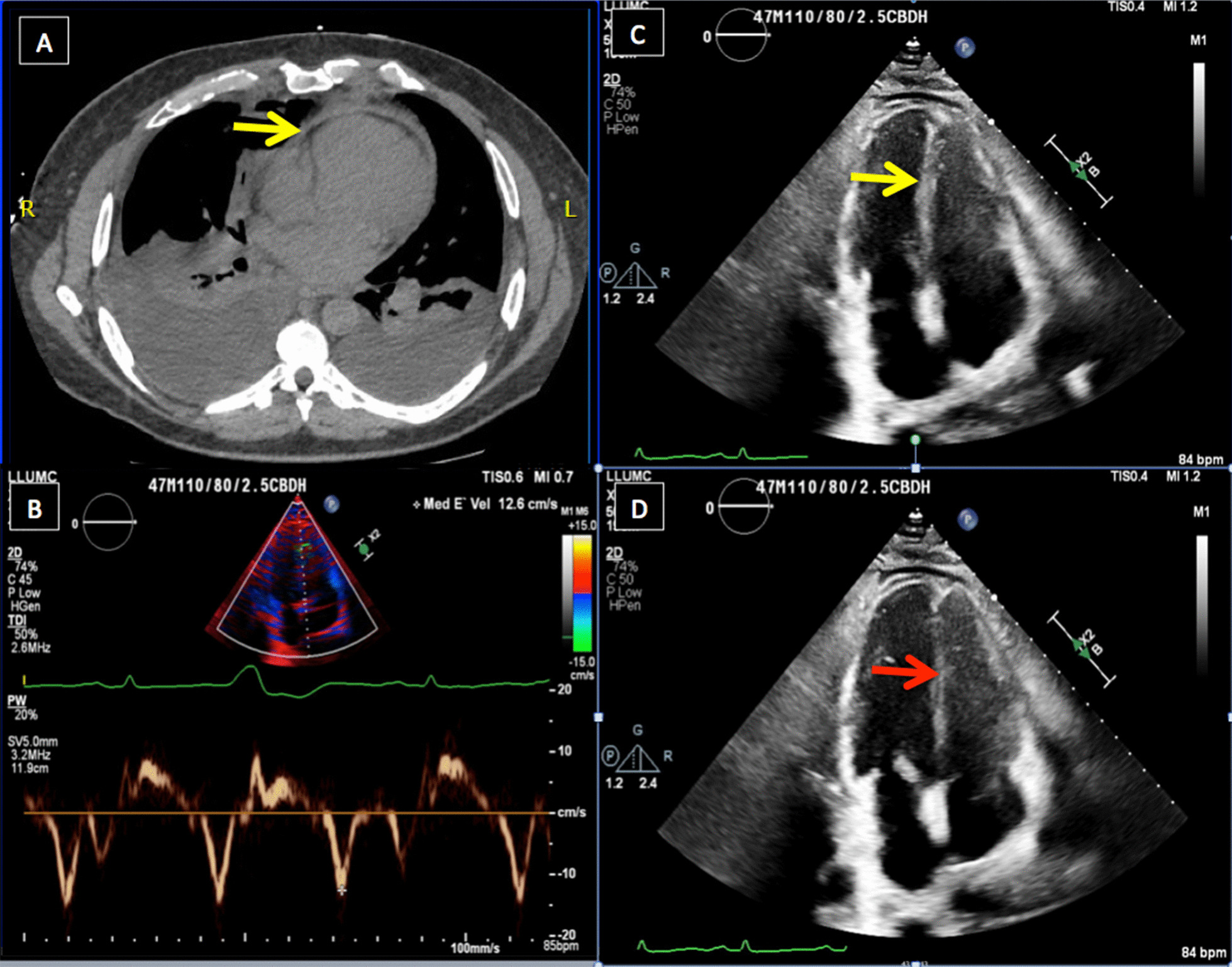
**a**, **b** Thickened pericardium in anterior–posterior (**a**) and lateral views (**b**). Black arrow identifies hemorrhage in pericardial space. White arrow points towards markedly thickened pericardium. **c**–**f** Hematoxylin and eosin stained histomicrographs of pericardium. **c** Scattered hemosiderin deposits are present within thickened and fibrotic pericardium consistent with prior hemorrhage (yellow arrows). **d** Diffuse, dense collagen deposition. **e** Extensive pericardial fibrosis resulting in markedly thickened and rough pericardial surface with adhesion formation. **f** Focus of normal-thickness pericardium shown for comparison

Through a median sternotomy hemorrhagic inflammation and tenacious adhesions were evident between the thickened pericardium and heart (Fig. 2a, b) requiring initiation of cardiopulmonary bypass using the femoral vessels. The pericardium was resected anteriorly and posteriorly from phrenic nerve to phrenic nerve. Separation from bypass, hampered by the development of RV dysfunction and profound vasoplegia, required significant pressor and inotropic support. Total bypass time was 144 min. He required plasma and platelets to obtain hemostasis. His central venous pressure dropped from 32 to 18 mmHg, the pulmonary artery systolic pressure was in the mid 20’s mmHg, his LV was vigorous and cardiac output remained 7–9 L/min.

**Fig. 3:**
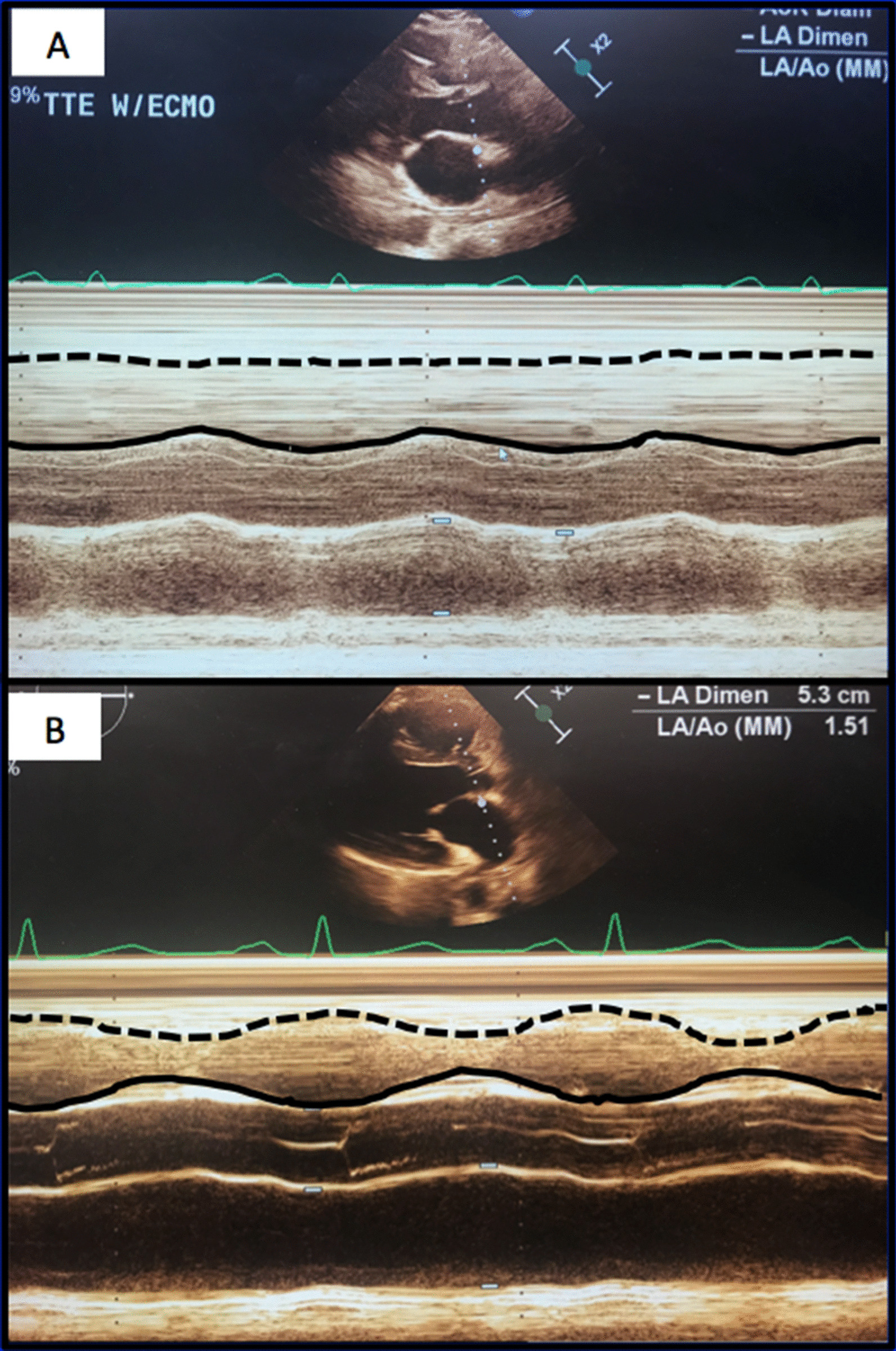
**a** M-mode transthoracic echocardiogram in parasternal long-axis demonstrating poor RV contractility in early postoperative period (**a**) and near normal RV contractility four months later (**b**). Dotted line represents RV free wall, solid line represents inter-ventricular septum

Over the next forty-eight hours, the vasoplegia worsened despite escalating pressor support. A transesophageal echocardiogram demonstrated akinesis of the RV (Fig. 3a). He developed renal failure requiring continuous renal replacement therapy. The next morning he was placed on femoral veno-arterial ECMO. Over the next week his pressor and inotropic support were weaned dramatically. He was decannulated after seven days support, extubated postoperative day 13 and transitioned to intermittent hemodialysis. His total bilirubin peaked at 32 mg/dL.

Over the next ten days his inotropic and pressor support were weaned off and his total bilirubin trended downwards. Repeat echocardiogram on day #23 showed mild to moderate RV hypokinesis. He was discharged to acute rehabilitation on day #25 and home ten days later. Pathologic examination (Fig. 2c–f) revealed a markedly thickened, fibrotic pericardium with chronic inflammation, fresh hemorrhage, old blood and significant adhesions.

Two months later his total bilirubin, renal function and weight had normalized. Four months after the original operation an echocardiogram showed mild RV dysfunction (Fig. 3b). One year later the patient felt well with no symptoms of heart failure but had persistent, unexplained lower extremity weakness. Of note, genetic testing subsequent to discharge confirmed the suspected diagnosis of HHT. The patient gave his permission for publication of this report.

## Discussion and conclusions

The impact of HHT on the management of constriction in this patient was most profound regarding the resulting bleeding diathesis likely compounded by the heparinization required for a longer than anticipated cardiopulmonary bypass run. The high transfusion requirement in theory could contribute to pulmonary congestion and high right ventricular afterload leading to RV failure but in this case the pulmonary pressures were normal. Right ventricular failure is a well-described cause of early morbidity and mortality after pericardiectomy [4]. Right ventricular deconditioning occurs over time due to myocardial atrophy from chronic under-filling of the chamber. The RV is supported by the pericardium that acts as an inelastic truss preventing dilatation. When the stiffened pericardium is removed the impaired RV has no further mechanical support, and abrupt increases in venous return can lead to volume overload, chamber dilation and RV failure.

In our patient ECMO was instituted on the second postoperative day, it allowed for de-escalation of pressor support and recovery of significant RV function. In retrospect, however, institution of ECMO more promptly may have allowed for a less prolonged recovery. Given the poor outcome of severe postoperative RV failure after pericardiectomy, we agree with previous recommendations [3] that with high central venous pressure, a low gradient between central venous and pulmonary artery pressures and high vasopressor requirements, that ECMO be instituted as soon as possible.

## Data Availability

Not applicable.

## Abbreviations

HHT: Hereditary hemorrhagic telangiectasia
ECMO: Extra-corporeal membrane oxygenation
LV: Left ventricle
RV: Right ventricle
